# Invest in adolescents and young people: it pays

**DOI:** 10.1186/1742-4755-10-51

**Published:** 2013-09-16

**Authors:** Venkatraman Chandra-Mouli, Rena Greifinger, Adaeze Nwosu, Gwyn Hainsworth, Lakshmi Sundaram, Sheena Hadi, Fran McConville, Regina Benevides, Callie Simon, Archana Patkar, Eva Schoening, Disha Sethi, Amy Boldosser-Boesch, Prateek Awasthi, Arvind Mathur, Doortje Braeken

**Affiliations:** 1 Scientist, Adolescent Sexual and Reproductive Health, Reproductive Health and Research, World Health Organization, Geneva, Switzerland; 2 Technical Advisor, Sexual and Reproductive Health and Tuberculosis Department, Population Services International, Washington, USA; 3 Health Policy and Management Student, Gillings School of Public Health, University of North Carolina at Chapel Hill, Chapel Hill, USA; 4 Senior Advisor, Adolescent Sexual and Reproductive Health, Pathfinder International, Watertown, USA; 5 Global Coordinator, Girls Not Brides: The Global Partnership to End Child Marriage, Aahung, Pakistan; 6 Technical Officer, Department of Maternal, Newborn, Child and Adolescent Health, WHO, Geneva, Switzerland; 7 Programme Manager, Networking and Knowledge Management, Water Supply and Sanitation Collaborative Council, Geneva, Switzerland; 8 Senior Advisor, Sexual and Reproductive Health and Rights to the organization, Deutsche Gesellschaft fuer Internationale Zusammenarbeit (GIZ), Geneva, Switzerland; 9 Project Coordinator, Digital Media and Learning, The YP Foundation, New Delhi, India; 10 Director, Global Advocacy, Family Care International, New York, USA; 11 Technical Analyst, United Nations Population Fund, New York, USA; 12 Medical Officer, Making Pregnancy Safer, WHO South East Asia Regional Office, New Delhi, India; 13 Senior Advisor, Adolescents and Young People, International Planned Parenthood Federation, London, England

## Abstract

This year’s Women Deliver conference made a strong call for investing in the health and development of adolescents and young people. It highlighted the unique problems faced by adolescent girls and young women–some of the most vulnerable and neglected individuals in the world–and stressed the importance of addressing their needs and rights, not only for their individual benefit, but also to achieve global goals such as reducing maternal mortality and HIV infection.

In response to an invitation from the editors of Reproductive Health, we-the sixteen coauthors of this commentary–put together key themes that reverberated throughout the conference, on the health and development needs of adolescents and young people, and promising solutions to meet them.

1. Investing in adolescents and young people is crucial for ensuring health, creating prosperity and fulfilling human rights.

2. Gender inequality contributes to many health and social problems. Adolescent girls and boys, and their families and communities, should be challenged and supported to change inequitable gender norms.

– Child marriage utterly disempowers girls. It is one of the most devastating manifestations of gender discrimination.

– Negative social and cultural attitudes towards menstruation constrain the lives of millions of girls. This may well establish the foundation for lifelong discomfort felt by girls about their bodies and reticence in seeking help when problems arise.

3. Adolescents need comprehensive, accurate and developmentally appropriate sexuality education. This will provide the bedrock for attitude formation and decision making.

4. Adolescent-centered health services can prevent sexual and reproductive health problems and detect and treat them if and when they occur.

5. National governments have the authority and the responsibility to address social and cultural barriers to the provision of sexual and reproductive health education and services for adolescents and young people.

6. Adolescents should be involved more meaningfully in national and local actions intended to meet their needs and respond to their problems.

7. The time to act is now. We know more now than ever before about the health and development needs of adolescents and young people, as well as the solutions to meeting those needs.

## Introduction

This year’s Women Deliver conference made a strong call for investing in the health and development of adolescents and young people. It highlighted the unique problems faced by adolescent girls and young women–some of the most vulnerable and neglected individuals in the world–and stressed the importance of addressing their needs and rights, not only for their individual benefit, but also to achieve global goals such as reducing maternal mortality and HIV infection.

The three-day conference was held in Kuala Lumpur, Malaysia in May 2013. It featured 700 presentations in more than 160 sessions and was attended by 4500 participants from 149 countries. The conference called for action to pave a future, for 2015 and beyond, that holds the health and well-being of girls and women as top priorities.

Adolescents and young people were center-stage at this year’s event, unlike in the two previous ones. Their health and development needs were discussed in dozens of sessions on different topics. And they were a notable physical presence. In addition to a youth pre-conference that brought together one hundred young leaders from around the world, adolescents and young people spoke on panels, moderated discussions, and chaired a youth networking zone.

In response to an invitation from the editors of Reproductive Health, we-the sixteen coauthors of this commentary–put together key themes that reverberated throughout the conference, on the health and development needs of adolescents and young people, and promising solutions to meet them. (All of us are passionate about young people; and two of us are young people).

### Investing in adolescents and young people is crucial for ensuring health, creating prosperity and fulfilling human rights

There are an estimated 1.2 billion adolescents (10–19 years old) in the world today; the largest population of adolescents in the history of mankind [1].

Adolescents and young people are a heterogeneous group–in different stages of development, living in different circumstances and with differing and changing needs. While some have the knowledge, understanding and skills to care for themselves and access support when they need it, many others are neither equipped nor supported to make the transition from childhood to adulthood. Further, some adolescents are especially vulnerable because they have physical or mental disabilities, engage in behaviours that are illegal or stigmatized, or are growing up in homes and communities where they are neither protected nor nurtured.

There has been substantial investment over many years in child survival resulting in impressive declines in childhood mortality [2]. However, the global health community has been slow to respond to the pressing needs of adolescents. There are sound reasons for continuing to invest in children, as they enter the second decade of life.

Investing in adolescent health will help prevent the estimated 1.4 million deaths that occur globally every year in this population group, due to road traffic injuries, violence and pregnancy-related causes. It will also improve the health and wellbeing of many millions of adolescents who experience mental health problems, nutritional deficiencies and the physical and psychosocial challenges associated with HIV infection. Investing in health promotion activities among adolescents now, such as anti-smoking and healthy eating initiatives, could yield huge returns in reducing the occurrence of non-communicable diseases such as lung cancer and diabetes in later life. Finally, investing in adolescent health can prevent problems in the next generation such as prematurity and low birth weight in children born to very young mothers [3].

Beyond public health, there are good economic reasons to invest in adolescent health as the World Bank stressed in its World Development Report in 2007 [4]. Broadening opportunities for adolescents and young people to develop skills and use them productively, helping them acquire the capabilities to make good decisions in pursuing those opportunities, and offering them second chances to recover from bad decisions, either their own or others–will ensure that they are a valuable resource and not an economic burden or threat to social harmony [5]. As signatories to the Convention on the Rights of the Child, countries have an obligation to meet the needs and fulfill the rights of adolescents and young people, especially those who are marginalized and are more likely to experience health and social problems.

### Gender inequality contributes to many health and social problems. Adolescent girls and boys, and their families and communities, should be challenged and supported to change inequitable gender norms

Gender and social expectations shape how adolescents think about themselves and others, and how they relate to members of the same and opposite sex. Deeply ingrained gender roles and unequal power relationships hinder the ability of girls and young women to refuse unwanted sex, negotiate condom use, make contraceptive choices, and discuss family planning and child spacing with their partners. Gender inequality also affects girls’ and women’s ability to seek and obtain the health services they need, leaving many to face serious health consequences [6].

Addressing gender inequality and gender-based violence must start with naming, challenging and changing these negative gender norms and building norms that value girls and boys, women and men equally. Approaches that have shown promise are those that promote positive norms around masculinity, and engage boys and girls in activities that foster reflection and dialogue about gender inequality. They aim to get individuals, groups and communities to challenge stereotypes and adopt more equitable norms. Such approaches can be combined with sexuality education and life skills building. Boys and men must be fully involved; they must be challenged and supported to be part of the solution [6].

### Box 1: Child marriage utterly disempowers girls. It is one of the most devastating manifestations of gender discrimination

Child marriage is a widespread harmful practice that affects enormous numbers of girls. It denies girls opportunities to study and to build livelihood skills. In addition it increases their vulnerability to health and social problems.

Child marriage persists because of poverty, low levels of education and social norms that families feel pressured to conform to; and because laws that forbid child marriage can be circumvented or ignored. In some places it is seen as a form of protection for girls. The reality is that girls who are married as children are particularly at risk of violence and abuse within marriage. They are often cut off from their families and friends. They are under pressure to bear children. And they are not reached by programmes targeting either adolescents or adult women. Even when they are, these services do not address the unique challenges that they face [7].

Child marriage warrants attention as a public health issue, a women’s health issue and as a human rights issue. We need to invest more in policies and programmes to prevent child marriage, both to reduce child and maternal deaths and illnesses, and to protect the rights of girls [7].

There is compelling evidence that a combination of strategies, tailored to local realities, can prevent child marriage. These include providing girls with information about their health and rights, building their skills and strengthening their links to networks; enrolling and retaining them in school; raising awareness of the negative effects of child marriage among their families and communities, and mobilizing them to take a stand against child marriage; providing economic incentives to girls and families so they do not consider marriage as a financial option; and enacting and enforcing laws to prevent child marriage [8].

### Box 2: Negative social and cultural attitudes towards menstruation constrain the lives of millions of girls. This may well establish the foundation for lifelong discomfort felt by girls about their bodies and reticence in seeking help when problems arise

In many low and middle income countries, negative attitudes towards menstruation constrain the lives of millions of girls. This may also establish the foundation for life-long disempowerment. In many countries, girls are unaware and unprepared for menarche. In places like Nepal, menstrual blood is believed to be polluted; as a result, girls and women are often forbidden from cooking, praying and participating in social activities during their periods. They may also be prohibited from eating certain foods and bathing. Young women working in garment factories in Bangladesh use bits of rags from the clothes they stitch, as make-shift sanitary pads. Because many schools across rural Africa do not have functional toilets or running water, girls have no option but to stay at home during their periods. In northern Tanzania for example, this has resulted in girls failing to fulfill the required school attendance days, and hence being unable to sit their exams. Ironically, this means that disenfranchisement for so many girls begins in school [9].

A comprehensive approach is needed to tackle the various challenges presented by menstruation. At the individual level, girls and boys need to be educated about puberty. At the family level, girls need support during their menstrual cycles. At the community level, we need to find ways to improve access to sanitary products, running water, functional toilets and privacy. We need competent and caring health care workers who can respond to girls’ questions and concerns, and provide care when they have menstrual health problems. Finally, we need leaders who can change the perception of menarche and menstruation to one of promise and pride, rather than of shame. These actions combined can build girls’ self-esteem, enable them to take greater charge of their lives, and create safe and supportive environments for them to grow and thrive [9].

### Adolescents need comprehensive, accurate and developmentally appropriate sexuality education. This will provide the bedrock for attitude formation and decision making

Improving adolescents’ knowledge and understanding of sexual and reproductive health, including HIV/AIDS, and building their life-skills to take charge of their health, is a crucial step in meeting their health needs and fulfilling their rights.

As boys and girls move from older childhood into and through adolescence, and then into early adulthood, they need sexuality education that responds to their developmental stages and circumstances and evolves with their evolving needs [10].

In many contexts, adolescents and young people are ill-informed about their bodies and their health, and unprepared for the changes they are experiencing and challenges they could face. They receive mixed messages. On the one hand, their parents and teachers tell them not to have sex before marriage, to smoke or to consume alcohol. On the other hand, they are flooded with images and messages in the popular media that associate these very behaviours with glamour and adventure, and suggest to them these behaviours are normal among their peers [10].

Involving adolescents in the design and delivery of sexuality education can help ensure that messages match their needs and concerns, appeal to them, and reach them through channels that they use [10].

### Adolescent-centered health services can prevent sexual and reproductive health problems and detect and treat them if and when they occur

Sexual and reproductive health services can enable adolescents to avoid health problems, and can enable those who experience health problems to get back to good health. In many countries, adolescents and young people are unable to obtain the health services they need. For example, restrictive laws and policies forbid the provision of contraceptives to unmarried adolescents in many places. Even when they are able to obtain health services, they are often reluctant to do so because of fears about privacy and confidentiality, and of being judged. When they do seek care, many adolescents and young people say that they are treated with disrespect or are even turned away by providers who refuse to offer them services [10].

A variety of approaches are being used to make health services welcoming and responsive to adolescents and young people. In some cases, health facilities (especially those run by nongovernment organizations) are purpose-built for them. In others, health workers that primarily work with all segments of the population are being trained and supported, and health facilities reoriented, to respond to the special needs and preferences of adolescents and young people [10].

Increased resources and support are needed to reach large numbers of adolescents and young people in countries where human and financial resources are scare. In the run-up to the Millennium Development Goals target date, many countries are strengthening their family planning, maternal health and HIV programmes. Making these services adolescent-centered, provides a real opportunity to reach the many adolescents who need preventive and curative services [11].

Finally, setting sexual and reproductive health service provision and sexuality education within a broader developmental approach aimed at improving access to education and employment provides the best promise for adolescent health and development.

### Box 3: We have an obligation to provide adolescents with contraceptive services that are responsive to their needs and preferences, and respectful of them

Substantial numbers of adolescents experience the negative health consequences of early, unprotected sexual activity-unintended pregnancy, unsafe abortions, pregnancy-related mortality and morbidity and STI including HIV; as well as its social and economic costs.

Adolescents in many places have serious misconceptions about contraception and are unable or unwilling to obtain and use them.

From research studies and projects there is compelling evidence of effective ways of delivering contraceptive information and services to different groups of adolescents in a variety of resource-constrained settings. But in most low and middle income countries adolescents find it very difficult to obtain contraceptives [12].

To meet the needs and fulfill the rights of adolescents, countries should eliminate medical and social restrictions to the provision of contraceptives to adolescents, and support and enable adolescents to obtain contraceptive methods that are appropriate to their needs and preferences through delivery mechanisms that are acceptable to them. Programmes must understand and respond to different groups of adolescents e.g. those who are unmarried, those who have given birth or had an abortion, and those who are living with HIV.

### National governments have the authority and the responsibility to address social and cultural barriers to the provision of sexual and reproductive health education and services for adolescents and young people

Almost universally, social and cultural barriers hinder the provision of comprehensive sexuality education and sexual and reproductive health services for adolescents and young people. Understanding and addressing these barriers is crucial.

Engaging and dialoguing with influential people and institutions can help generate community and society level support for the provision of sexual and reproductive health education and services. National governments have the authority and the responsibility to do this, and international organizations have a responsibility both to support governments and to press for accountability.

Culture and tradition are sometimes used to justify the continuation of practices that have lasting negative consequences such as child marriage and female genital mutilation. They are also used to withhold sexual and reproductive health education and services.

As the main duty bearers, governments are obliged to protect children from harm and support their development in line with the Convention on the Rights of the Child. National governments should take culture and tradition into account as their develop policies and strategies and implement programmes, but should not allow them to be used as an excuse to withhold these rights.

### Adolescents should be involved more meaningfully in national and local actions intended to meet their needs and respond to their problems

Adolescents and young people are at the forefront of political movements for change throughout the world. Their outrage, their passion and their fearlessness is evident from the streets of New Delhi, to Rio, and to Cairo. In these places and elsewhere, they are creating real change.

Knowledgeable, articulate and confident young people are making their voices heard in global events and discourses. However, we need to do more to tap into their energy, and draw upon their intimate knowledge of their worlds to guide policy and practice on health and development. Young people have shown that they can make useful contributions as peer educators and motivators, and as distributors of educational materials and health products such as condoms. They have also shown that when appropriate mechanisms are in place, they play a vital role in formulating policies, and in designing, implementing, monitoring and evaluating programmes and projects that address their needs and problems [13].

### The time to act is now

We know much more today about what adolescents and young people need to grow and develop in good health. We know much more about the health and social problems they face, and more importantly about which groups of adolescents are more likely to experience these problems. Finally, we know much more about effective interventions to prevent these problems and to respond to them when they occur. While we need to continue our research to add to our knowledge and understanding, the real imperative is to apply the knowledge and understanding that we already have.

There is widespread acceptance of the need to address the sexual and reproductive health of adolescents and young people. There is a groundswell of support from national and international bodies to translate words into action. We need to leverage this collective commitment and expertise. For the world’s 1.2 billion adolescents to survive, grow and develop to their full potential, the small scale, time limited, piecemeal projects of yesterday must be transformed into the strong, large scale and sustained programmes of today.

## Competing interests

The authors declare that they have no competing interests.

## Authors’ contributions

VC conceived the paper. All the authors provided inputs. VC prepared the drafts, shared them with the authors, collated the feedback and prepared revised drafts. AN assisted him with this work. Many-but not all-the authors provided feedback on the successive drafts. All the authors read and approved the final draft.
